# Dopamine Release from the Locus Coeruleus Enables Temporal Binding in the Hippocampus

**DOI:** 10.64898/2026.10.13.758657

**Published:** 2026-10-15

**Authors:** Ana Paula Crestani, Cassiano Ricardo Alves Faria Diniz, Deger Carda, Savannah Maw, Brian Joseph Wiltgen

## Abstract

The hippocampus forms memories by associating events that are separated in time. These associations can be studied using trace conditioning, a procedure where a temporal gap is inserted between a conditional stimulus and an unconditional stimulus. Here, we examined how dopaminergic projections to CA1 support trace fear conditioning. Using fiber photometry and genetic lesions, we demonstrate that locus coeruleus (LC)-derived dopamine, rather than VTA input, is the primary source of dopamine in CA1 and is essential for trace learning. LC lesions reduced CA1 principal neuron activity and c-Fos expression. In-vivo imaging revealed that successful learning requires post-shock reactivation of stimulus-specific ensembles, a process compromised by LC lesions or long trace intervals. Finally, optogenetic stimulation of LC-CA1 projections during the post-shock period was sufficient to rescue learning in long-trace protocols. These findings identify LC-CA1 dopamine as a critical regulator of temporal binding via the facilitation of hippocampal memory trace reactivation.

